# Secreted Chaperones in Neurodegeneration

**DOI:** 10.3389/fnagi.2020.00268

**Published:** 2020-08-27

**Authors:** Kriti Chaplot, Timothy S. Jarvela, Iris Lindberg

**Affiliations:** Department of Anatomy and Neurobiology, University of Maryland School of Medicine, University of Maryland, Baltimore, Baltimore, MD, United States

**Keywords:** proteostasis, neurodegeneration, sHsp, clusterin, BRICHOS, 7B2, proSAAS, progranulin

## Abstract

Protein homeostasis, or proteostasis, is a combination of cellular processes that govern protein quality control, namely, protein translation, folding, processing, and degradation. Disruptions in these processes can lead to protein misfolding and aggregation. Proteostatic disruption can lead to cellular changes such as endoplasmic reticulum or oxidative stress; organelle dysfunction; and, if continued, to cell death. A majority of neurodegenerative diseases involve the pathologic aggregation of proteins that subverts normal neuronal function. While prior reviews of neuronal proteostasis in neurodegenerative processes have focused on cytoplasmic chaperones, there is increasing evidence that chaperones secreted both by neurons and other brain cells in the extracellular – including transsynaptic – space play important roles in neuronal proteostasis. In this review, we will introduce various secreted chaperones involved in neurodegeneration. We begin with clusterin and discuss its identification in various protein aggregates, and the use of increased cerebrospinal fluid (CSF) clusterin as a potential biomarker and as a potential therapeutic. Our next secreted chaperone is progranulin; polymorphisms in this gene represent a known genetic risk factor for frontotemporal lobar degeneration, and progranulin overexpression has been found to be effective in reducing Alzheimer’s- and Parkinson’s-like neurodegenerative phenotypes in mouse models. We move on to BRICHOS domain-containing proteins, a family of proteins containing highly potent anti-amyloidogenic activity; we summarize studies describing the biochemical mechanisms by which recombinant BRICHOS protein might serve as a therapeutic agent. The next section of the review is devoted to the secreted chaperones 7B2 and proSAAS, small neuronal proteins which are packaged together with neuropeptides and released during synaptic activity. Since proteins can be secreted by both classical secretory and non-classical mechanisms, we also review the small heat shock proteins (sHsps) that can be secreted from the cytoplasm to the extracellular environment and provide evidence for their involvement in extracellular proteostasis and neuroprotection. Our goal in this review focusing on extracellular chaperones in neurodegenerative disease is to summarize the most recent literature relating to neurodegeneration for each secreted chaperone; to identify any common mechanisms; and to point out areas of similarity as well as differences between the secreted chaperones identified to date.

## Introduction

Protein homeostasis, or proteostasis, is a combination of events that govern protein quality control, namely, protein translation, folding, processing and degradation. Disruptions in these events can lead to protein misfolding and aggregation. Proteostatic disruptions can arise normally as a consequence of normal aging or can be due to genetic mutations and environmental stressors such as heat shock, altered energy demands, or pH changes, and can lead to cellular changes such as ER and/or oxidative stress; organelle dysfunction; and even cell death. Protein misfolding and aggregation are central to several proteinopathies such as neurodegenerative disease, various amyloidosis, cystic fibrosis and sickle cell anemia, among others. Chaperones, ubiquitous proteins dedicated to the management of protein homeostasis, reside in every cellular compartment. For example, a well-studied class of abundant chaperones known as HSPs, are mostly cytoplasmic, are ATP-dependent, and often work in a protein network or in a sequential pathway (reviewed in Hartl et al., 2011; Klaips et al., 2018). Chaperones are responsible for binding nascent protein chains, preventing them from aggregating, and for promoting the formation of secondary and tertiary structures to obtain the stable conformations required for proper function. Chaperones also play a role in regulating active and inactive functional protein states, as well as in downstream protein processing events such as proteolytic cleavage and post-translational modification. Finally, chaperones are involved in unfolding and delivering proteins, working closely with ubiquitin ligase complexes and proteases, to degradative pathways.

### Players in Secretory Pathway Proteostasis

Typically, proteins destined for secretion pass through the membrane-bound secretory pathway of the cell. Upon or during translation, the pre-proteins are channeled into the ER with the help of the signal sequence. Following chaperone-assisted folding, proteins are then subject to further processing which includes posttranslational modifications, and/or proteolytic maturation cleavages. Protein modification occurs throughout the secretory pathway, across the ER, ER-Golgi intermediate compartment, Golgi complex, *trans*-Golgi network and finally, secretory vesicles. Once secreted into the extracellular matrix, proteins can be taken up by the neighboring cells through endocytosis and eventually subjected to lysosomal degradation. Throughout this process, chaperones resident within these various compartments aid in maintaining client protein stability and localization.

The extremely high protein concentrations present in neurons and endocrine cells, which possess both regulated (stimulus-dependent) and constitutive (basal) secretory pathways, are conducive to homo- and heterotypic aggregation, and ER-resident chaperones, which assist the retro-translocation of aggregated proteins to the cytosol, play an important role in preventing misfolded proteins from causing a bottleneck in the secretory pathway during stress. An example of an ER stress-related chaperone is the ER-localized protein BiP, a member of the HSP70 family. BiP chaperones ER-based proteins under normal conditions; however, in the presence of ER stress, it binds and retro-translocates misfolded proteins from the ER to the cytoplasm for proteasomal degradation as a member of the ERAD machinery (Pobre et al., 2019). In another example, evidence has emerged linking the UPR to the regulation of extracellular proteostasis. In response to ER stress, the chaperone HSP40 (a.k.a ERdj3) is co-secreted from the cell with unstable protein clients, which may assist in prevention of cytotoxic aggregation (Genereux and Wiseman, 2015b; Genereux et al., 2015a).

### Cytoplasmic Versus Secreted Chaperones: The Challenge of the Extracellular Space

The challenges faced by chaperones secreted from the cell are markedly different than those of cytoplasmic chaperones. While cytoplasmic chaperones deal with initial folding of nascent chains of secretory proteins (and unfolding during degradation), additional chaperone assistance is required during transport through the secretory pathway – not only for stabilizing protein folds, but also for proper localization of proteins in the secretory pathway compartments, as well as aiding their secretion into the extracellular matrix.

The extracellular space presents unique conditions such as low pH, low protein density and low ATP availability that affects client-chaperone interaction. The low pH of the secretory pathway and the extracellular space directly affect protein stability, necessitating chaperone action even under normal conditions. While the concentration of chaperones such as clusterin in the CSF is 30–50 nM [(Přikrylová Vranová et al., 2016); see section “Clusterin” below], the CSF concentration of other chaperones such as 7B2 is even lower, in the low nanomolar range (Iguchi et al., 1987a), rendering client interaction even more challenging. It may be speculated that the release of neuronally synthesized chaperones directly into the synaptic space alleviates this scarcity. Another challenge relates to the fact that cytoplasmic chaperones are mostly ATP-dependent, enabling them to bind hydrophobic regions of nascent proteins, and release folded proteins with ADP conversion. Owing to low extracellular ATP, secreted chaperones appear to act more as “holdases” rather than “foldases,” binding to exposed hydrophobic regions of misfolded proteins, thereby preventing protein self-assemblies and aggregation (reviewed in Wyatt et al., 2013) (Hartl et al., 2011; Klaips et al., 2018). Secreted chaperones may also deliver proteins to cellular membrane receptors that aid in their internalization and subsequent lysosomal degradation (Yerbury et al., 2005; Wyatt et al., 2013).

### Proteostasis in Neurodegeneration

A majority of neurodegenerative diseases involve the pathologic aggregation of proteins that subverts normal neuronal function (Soto and Pritzkow, 2018). These protein aggregates, which include plaques composed of beta amyloid peptides, Lewy bodies containing synuclein, tangles containing tau proteins, and cytoplasmic aggregates of triple-repeat proteins, represent physical hallmarks of neurodegenerative processes, and reflect aberrant protein handling mechanisms that are predominantly focused in neural tissues. These protein deposits arise from aggregation-prone proteins that often possess intrinsically disordered domains, allowing them to form self-replicating structures that tend to oligomerize and form fibrillary or non-fibrillary aggregates. The oligomeric species are most often found to be the toxic form that interferes with normal cellular function. Both extracellular and intracellular proteins are at risk of aberrant aggregation, and new data suggest that there is cross-talk between different disease processes such that “seeds” from one aggregating species can cause aggregate deposition of entirely different proteins (reviewed in Peng et al., 2020). The demonstration of transsynaptic propagation of a variety of misfolded proteins during neurodegenerative disease progression suggests that extracellular processes are critical to the process of neurodegeneration (Peng et al., 2020). The significant co-morbidity of diabetes with AD suggests a generalized risk to secretory tissues in neurodegenerative disease, potentially from common metabolic factors with increased susceptibility to errors in normal protein handling (Shieh et al., 2020). While most reviews of neuronal proteostasis in neurodegenerative processes have focused on cytoplasmic chaperones (Hartl et al., 2011; Kim Y.E. et al., 2013; Balchin et al., 2016), there is increasing evidence that chaperones secreted by neurons and other brain cells play important roles in neuronal proteostasis (reviewed in Wyatt et al., 2013).

In this review, we will summarize various secreted chaperones and describe the evidence for their involvement in neurodegenerative processes. We have necessarily omitted many secreted proteins which may in future turn out to contribute to proteostasis in neurodegenerative disease; examples include SPARC/osteonectin, which exhibits extracellular chaperone activity (Chlenski et al., 2011) and has repeatedly been linked to neurodegeneration (reviewed in Chen S. et al., 2020; Pedrero-Prieto et al., 2020). However, its general chaperone activity against neurodegeneration-related aggregates has not yet been documented. A variety of personal chaperones, for example, PCSK9 (proprotein convertase subtilisin/kexin 9), RAP (receptor-associated protein) and MESD (mesoderm development), all of which accompany LDLR family members through the secretory pathway, are not discussed, nor are the many other personal chaperones that accompany other secretory proteins to specific subcellular destinations, including COSMC (core 1 β3GalT specific molecular chaperone), PPCA (protective protein/cathepsin A), Praf/STMC6 (prenylated Rab acceptor family members), and the iRhom (inactive rhomboid) proteins. However, as found with the personal chaperones 7B2 and proSAAS, it is possible that future work will reveal that other secretory pathway personal chaperones have a wider client base than previously suspected.

We have also not discussed Hsps and other chaperone proteins resident within the secretory pathway which can under specialized stress circumstances be secreted, such as ERDj3 (Genereux et al., 2015a); other ER-resident chaperones (Trychta et al., 2018); cyclophilins (Hoffmann and Schiene-Fischer, 2014); or ERAD-associated proteins such as Stch/HSPA13 (Chen et al., 2009). While these proteins clearly function as chaperones within the secretory pathway, strong evidence for their secretion during neurodegeneration is still lacking (for example documented presence within extracellular aggregates and/or altered CSF levels associated with disease). Finally, in the interest of brevity, we do not discuss well-known secreted chaperones such as α-2-macroglobulin and transthyretin which, although abundant in serum and able to cross the blood–brain barrier, are not highly expressed by the brain, and have also been recently reviewed in the context of neurodegeneration (Buxbaum and Johansson, 2017; Cater et al., 2019; Giao et al., 2020).

The secreted chaperones described in this review have all been associated with neurodegenerative disease based on specific features which include, among others, their physical presence in protein deposits; and proteomics and transcriptomics studies from human as well as animal models that highlight altered levels in neurodegenerative disease. Many of these features are summarized in Table 1. The presence of a given chaperone within protein deposits is consistent with, but does not prove, its propensity to sequester aggregation-prone protein clients; it may, for example, simply be an easily aggregated bystander. In addition, altered extracellular or intracellular levels seen in disease might represent a disease-related response to combat excessive misfolding, but also might point to a generally dysregulated secretory pathway. However, the additional ability of the secreted chaperones discussed here to profoundly reduce the rate of protein oligomerization *in vitro* supports a likely role in regulating disease pathogenesis.

**TABLE 1:** Extracellular chaperones implicated in neurodegenerative diseases - Human studies.

Name(s)	Gene name(s)	Disease link		Location	Method	Citations
Clusterin, Apo-J, SP40/40, CLI	*CLU*	AD –	Associated with Abeta40 plaques	C	IF/IHC	Howlett et al. (2013)
			Genetic risk factor		GWAS	Harold et al. (2009), Lambert et al. (2009)
			Increased protein levels	CSF	MS	Reviewed in Pedrero-Prieto et al. (2020)
		PD –	Associated with Lewy bodies	C	IHC	Sasaki et al. (2002)
		CJD –	Associated with protein deposits	CB	IHC/IP	Freixes et al. (2004)
			Increased expression	C/CB	DNA micro-array	Freixes et al. (2004)
		ALS –	Associated with TDP43 inclusions	SC	IF/IHC	Gregory et al. (2017)
			Increased protein levels	Serum	MS	Xu et al. (2018)
Progranulin	*GRN*	AD –	Associated with Abeta plaques	HC	IF/IHC	Gliebus et al. (2009), Gowrishankar et al. (2015), Mendsaikhan et al. (2019a)
			Genetic risk factor		GWAS	Chen et al. (2015)
			High protein levels	CSF	ELISA	Suárez-Calvet et al. (2018)
		FTD –	Null mutations		Linkage analysis	Baker et al. (2006)
			Decreased protein levels	CSF	WB	Wilke et al. (2017)
Bri2, Bri3	*ITM2B, ITM2C*	AD –	Associated with Abeta40 and Abeta42 plaques	HC	IF/IHC	Del Campo et al. (2014), Dolfe et al. (2018)
		FBD and FDD –	Read-through mutations causing protein deposits	C	IF/IHC	Reviewed in Rostagno et al. (2005)
ProSAAS	*PCSK1N*	AD –	Associated with Abeta plaques	HC	IF/IHC	Hoshino et al. (2014)
			Decreased protein levels	CSF	MS	Abdi et al. (2006), Finehout et al. (2007), Jahn et al. (2011), Choi et al. (2013), Holtta et al. (2015), Spellman et al. (2015)
			Increased mRNA levels	C	RNA-seq	Mathys et al. (2019)
		PD –	Associated with Lewy bodies	HC	IF	Jarvela et al. (2016)
			Decreased protein levels	CSF	MS	Rotunno et al. (2020)
		FTD –	Decreased protein levels	CSF	MS	Davidsson et al. (2002)
		DLB –	Decreased protein levels	CSF	MS	Van Steenoven et al. (2020)
7B2	*SCG5*	AD –	Associated with plaques	HC	IF/IHC	Helwig et al. (2013)
			Slightly increased protein levels	C	WB	Iguchi et al. (1987a)
		PD –	Associated with Lewy bodies		IF/IHC	Helwig et al. (2013)
		ALS –	Increased protein levels	CSF	MS	Ranganathan et al. (2005)
		FTD –	Increased protein levels	CSF	MS	Mattsson et al. (2008)
HspBl	*HSPB1*	CMT –	Causative mutations		GWAS, NGS	Muranova et al. (2019), Vendredy et al. (2020)
		AD –	Associated with plaques	HC	IF/IHC	Wilhelmus et al. (2006), Ojha et al. (2011)
		PD –	Increased protein levels	C	IF/IHC	Renkawek et al. (1999)
HspB3	*HSPB3*	CMT –	Causative mutations		GWAS, NGS	Muranova et al. (2019), Vendredy et al. (2020)
		AD –	Associated with plaques	HC	IF/IHC	Wilhelmus et al. (2006), Ojha et al. (2011)
			Increased protein levels	HC, C, CG	MS	Koopman and Rüdiger (2020)
		MS –	Increased protein levels	Serum	WB	Ce et al. (2011)
HspB5	*HSPB5*	AD –	Increased protein levels	HC, C, CG	MS	Koopman and Rüdiger (2020)
HspB6	*HSPB6*	AD –	Increased protein levels	HC, C, CG	MS	Koopman and Rüdiger (2020)
HspB8	*HSPB8*	CMT –	Causative mutations		GWAS, NGS	Muranova et al. (2019), Vendredy et al. (2020)
		AD –	Associated with plaques	HC	IF/IHC	Wilhelmus et al. (2006), Ojha et al. (2011)
			Increased protein levels	HC, C, CG	MS	Koopman and Rüdiger (2020)
		CAA –	Associated with amyloid plaques	C	IF/IHC	Wilhelmus et al. (2009)

*AD, Alzheimer’s disease; ALS, amyotrophic lateral sclerosis; CAA, cerebral amyloid angiopathy; CMT, Charcot-Marie-Tooth; DLB, dementia with Lewy bodies; FBD, familial British dementia; FDD, familial Danish dementia; FTD, frontotemporal dementia; CJD, Creutzfeldt–Jakob disease; PD, Parkinson’s disease; C, cortex; CB, cerebellum; CG, cingulate gyrus; CSF, cerebrospinal fluid; HC, hippocampus; SC, spinal cord; ELISA, enzyme-linked immunosorbent assay; GWAS, genome wide association study; IHC, immunohistochemistry; IF, immunofluorescence; IP, immunoprecipitation; NGS, next generation sequencing; MS, mass spectrometry; RNA-seq, RNA sequencing; WB, Western blot.*

In the following sections, select chaperones will be described that have been clearly identified as causative agents in neurodegenerative diseases: clusterin, progranulin, and BRICHOS domain-containing proteins. Next, the neural and endocrine-specific proteins, 7B2 and proSAAS, are discussed with respect to their potent anti-aggregant activity and association with neurodegenerative disease. We will then focus on extracellular actions of certain secreted small Hsps (sHsps); while sHsps have been extensively reviewed recently, most reviews in neurodegeneration have focused on their cytoplasmic actions (Muranova et al., 2019; Webster et al., 2019). Finally, we will elaborate on possible biochemical and physiological mechanisms of extracellular chaperones and discuss their therapeutic potential in treating neurodegenerative disease.

## Clusterin

Clusterin is a heavily glycosylated ∼60 kDa heterodimeric protein (Kapron et al., 1997) derived from a single gene, known as *CLU*, which represents both a potent as well as a highly abundant extracellular ATP-independent chaperone. It is able to block Abeta aggregation (Matsubara et al., 1996) as well as amyloid formation from a variety of amyloidogenic substrates (Yerbury et al., 2007) and has the ability to prevent stress-induced precipitation of a wide range of protein substrates (Humphreys et al., 1999; Poon et al., 2000). This versatile chaperone plays many roles in homeostatic processes throughout the body, including lipid transport (reviewed in Park et al., 2014), tissue remodeling (Gobé et al., 1995), cytoprotection at fluid/tissue interfaces (reviewed in Fini et al., 2016), and endocytosis-mediated clearance of extracellular debris (Bartl et al., 2001). Additionally, increased clusterin expression is linked to cancer progression and treatment resistance (reviewed in Peng et al., 2019). This versatility is thought to derive from clusterin’s ability to interact with a variety of different client misfolded proteins. As discussed below, clusterin accumulation is found in many different neurodegenerative diseases.

### Structure and Expression

Clusterin is expressed in many cell types throughout the body, most notably in specialized secretory cells and epithelial cells (Aronow et al., 1993). Within the brain, clusterin is ubiquitously expressed in neurons and glia, and is especially abundant in astrocytes, while being absent from microglia (Yao et al., 2020)^1^. Circulating clusterin levels are very high in serum, predominantly derived from the liver (Seo et al., 2020), and approximate 100 μg/ml (about 1.6 μM). Although at much lower levels than in plasma, clusterin is also abundant in the CSF, with normal levels between 2–9 μg/ml (30–150 nM) (Sihlbom et al., 2008; Přikrylová Vranová et al., 2016).

Within the cell, as a signal-bearing protein, clusterin exists primarily within the secretory pathway. Under various types of stress conditions, cellular clusterin levels are increased (Viard et al., 1999), and a portion of this increase may be within the cytoplasm and/or nucleus (Nizard et al., 2007; Prochnow et al., 2013). However, the origins of cytoplasmic and nuclear clusterin are still unclear (reviewed in Rohne et al., 2016) since current hypotheses to explain these subcellular locations are discordant. Cytoplasmic clusterin may arise from *CLU* transcripts translated directly into the cytosol; from secretory clusterin that has prematurely exited the secretory pathway; or from reuptake of secreted mature clusterin (discussed in Foster et al., 2019). Alternatively, intracellular and nuclear clusterin may be produced from rare distinct mRNA transcripts that arise from alternative splicing and different in-frame start sites (reviewed in Garcia-Aranda et al., 2018). However, these transcripts make up less than 0.34% of total clusterin mRNA (Prochnow et al., 2013), suggesting that the vast majority of clusterin protein is produced from the primary transcript. *CLU* expression is affected by histone acetylation, DNA methylation, and a variety of transcription factors and signaling molecules (Garcia-Aranda et al., 2018). Additionally, clusterin expression responds to oxidative and proteotoxic stress, through heat shock transcription factor-1 and activator protein-1 elements in the *CLU* promoter (reviewed in Trougakos, 2013).

For secreted clusterin, intracellular cleavage of the clusterin precursor results in the production of α- and β-subunits arranged in an anti-parallel fashion, linked by five disulfide bridges. Heavy N-linked glycosylation accounts for ∼30% of its apparent mass (reviewed in Wilson and Easterbrook-Smith, 2000). Clusterin contains five conserved putative amphipathic helices, with a large percentage of intrinsically disordered structure (Bailey et al., 2001). The anti-parallel organization of the two chains creates a polarized order-to-disorder motif within the entire protein (Bailey et al., 2001) similar to that of a partially folded, molten globule-like domain capable of interacting with a wide variety of ligands. Clusterin has been shown to exist in various pH-dependent multimeric states (Hochgrebe et al., 2000).

### Function and Mechanism

Of the three major functions that protein chaperones carry out (assisting folding/refolding, preventing precipitation/aggregation, and preparation for degradation), clusterin has been implicated in the latter two. In 1999, clusterin was shown to be a proficient solubilizing chaperone for heat-stressed glutathione-*S*-transferase by the Wilson group, exceeding the client:chaperone molar ratios of sHSPs (Humphreys et al., 1999). In the last two decades, clusterin has been found to bind and solubilize a variety of heat-stressed proteins (Poon et al., 2002), as well as to prevent the aggregation and fibrillation of disease-associated aggregating proteins, including: Abeta (Yerbury et al., 2007), α-synuclein (Yerbury et al., 2007), TAR DNA-binding protein 43 (TDP-43) (Gregory et al., 2017), and transthyretin (Lee et al., 2009; Magalhães and Saraiva, 2011). Single-molecule characterization has shown that clusterin can bind small Abeta40 oligomers at equimolar ratios (Narayan et al., 2011); small α-synuclein oligomers at equimolar ratios; and large oligomers at sub-stoichiometric ratios (Whiten et al., 2018). Clusterin binding also appears to inhibit oligomer interaction with lipid membranes and the production of reactive oxygen species (Whiten et al., 2018). However, at low molar ratios of clusterin to Abeta, clusterin promotes precipitation and increases fibrillation (Yerbury et al., 2007). *In vitro*, clusterin can sequester Aβ oligomers of various sizes (Narayan et al., 2011). Taken together, these data show that clusterin is capable of sequestering small oligomers, preventing both assembly into fibrils as well as blocking downstream toxic events.

*In vivo*, clusterin promotes transcytosis of Abeta across the blood–brain barrier through [lipoprotein-related protein 2 (LRP2)-mediated endocytosis] (Zlokovic, 1996; Wojtas et al., 2017). More broadly, clusterin has been shown to promote internalization and degradation of cellular debris and misfolded proteins in non-professional phagocytic cells through LRP2 (Bartl et al., 2001) and through HSPGs (Itakura et al., 2020). Itakura et al. (2020) demonstrated that clusterin binds HSPGs through an electrostatic interaction to promote co-degradation of clusterin and client proteins. HSPGs are broadly expressed and have been previously linked to the endocytosis of a variety of ligands (reviewed in Sarrazin et al., 2011), including Abeta (Kanekiyo et al., 2011), among others. Whether this endocytic pathway represents a major functional contribution of clusterin in neurodegenerative diseases remains to be established.

### Disease Relevance and Therapeutic Potential

Clusterin immunoreactivity has been identified within a variety of proteopathic aggregates, including amyloid plaques (Howlett et al., 2013; Craggs et al., 2016), perivascular amyloid deposits (Craggs et al., 2016), cortical Lewy bodies (Sasaki et al., 2002), and protease-resistant prion protein deposits in Creutzfeldt-Jakob disease (Freixes et al., 2004). Clusterin shows a preference for colocalization with Abeta40 over Abeta42 (Howlett et al., 2013; Craggs et al., 2016). Clusterin levels are increased at synapses in human post-mortem AD brains, where it colocalizes with Abeta at presynapses near plaques (<10 μm). This increase is greater in *APOE4* carriers (Jackson et al., 2019), and may be related to the increase in intracellular clusterin levels observed after Abeta-induced stress (Killick et al., 2014).

Considerable genetic evidence connects clusterin to neurodegenerative disease. After *APOE* and *BIN1*, *CLU* is the third largest genetic risk factor for late onset AD. Multiple GWAS (Harold et al., 2009; Lambert et al., 2009) and meta-analyses (Liu et al., 2014; Zhu et al., 2018) have confirmed a strong correlation of the rs11136000T allele with decreased AD risk in Caucasian populations; however, the evidence for this genetic link is weaker in other ethnic groups (Han et al., 2018). This protective allele is associated with increased clusterin expression (Ling et al., 2012). Additional rare polymorphisms have been identified in patients suffering from late onset AD, in which secreted clusterin levels are reduced due to folding abnormalities (Bettens et al., 2015). Six independent proteomics studies show that CSF levels of clusterin are significantly increased in AD patients (see meta-analysis in Pedrero-Prieto et al., 2020); plasma levels also rise in ALS (Xu et al., 2018). Additionally, while brain clusterin levels increase within the brain during AD progression, the levels of Abeta increase to a greater extent, resulting in a declining molar ratio between clusterin and Abeta specifically within regions of the brain with high Abeta deposition (Miners et al., 2017). Thus, while assessing clusterin levels in CSF may eventually prove to be a powerful diagnostic tool, these data suggest that brain clusterin levels will need to be increased to a great extent in order to provide a therapeutic benefit.

Animal studies of clusterin expression in neurodegenerative disease have yielded paradoxical results. When crossed with clusterin null mice, PDAPP AD model mice (which express the human APP mutant V717F at ten times the level of endogenous APP) show reduced levels of neuritic dystrophy and fibrillary amyloid plaques within the brain, without an overall change in levels of Abeta (DeMattos et al., 2002). Thus, this phenotype exhibits less neuritic toxicity than in APP model mice expressing clusterin. Another group who performed the same clusterin null cross with a different APP mouse model (APP/PS1; PS1 = presenilin1), which expresses lower levels of a mouse/human chimeric *APP* transgene found a reduction in fibrillar Abeta plaques concomitant with a reduced Abeta load (Wojtas et al., 2017). In this mouse model, Abeta deposits are found as increased CAA deposits, and overall Abeta load is decreased within the brain (Wojtas et al., 2017). These paradoxical results were corroborated by another group who showed that clusterin expression is required for Abeta toxicity in human induced pluripotent stem cell (iPSC)-derived neurons (Robbins et al., 2018). In agreement, in primary neurons, siRNA silencing of clusterin expression provides protection against Abeta toxicity (Killick et al., 2014). However, as shown by Robbins et al. (2018), the loss of clusterin expression results in a change in the expression of a variety of genes; thus, it is currently unclear if the paradoxical effect is directly related to clusterin loss or is due to pleiotropic effects. Adding to this complexity, in the 5xFAD (familial Alzheimer’s disease) model mouse background, clusterin null mice showed fewer Abeta plaques and cognitive performance deficits compared to clusterin-expressing mice at 5 months of age, though the differences disappeared by 10 months, suggesting that clusterin expression is only required for early toxicity (Oh et al., 2019). We speculate that the variation in results when using different mouse models may be due at least in part to differences in the clusterin: Abeta ratio inherent in the different experimental models. Alternative explanations for the positive effects of clusterin null mutations on neurodegenerative pathology include altered expression of other genes, as observed in Robbins et al. (2018), and thus this paradox remains unexplained.

Despite these controversial mouse studies, substantial biochemical evidence supports the positive effects of clusterin expression on Abeta load within the brain. Thus, several groups have directly examined the effects of increased brain clusterin on amyloid deposition in various mouse models of AD. Peripheral administration of recombinant human clusterin, either in complex with HDL or lipid-free, was shown to reduce the levels of insoluble Abeta as well as CAA in AD model mice (de Retana et al., 2019). Neuronal loss in the hippocampus was also decreased; whether this positive effect was due to the reduced damage from Abeta accumulation, or to clusterin’s known anti-apoptotic effects (Koch-Brandt and Morgans, 1996) is unclear. One caveat to this study is that recombinant clusterin was not detected within the brain, though it was found in the intrameningeal ventricle lumen. This is consistent with studies that show that while clusterin can cross the blood–brain barrier through interaction with LRP2 (Zlokovic, 1996), plasma levels of endogenous clusterin are sufficient to saturate the transporter (Shayo et al., 1997). These data suggest that the protective effects provided by recombinant clusterin are greater than simple blockade of Abeta accumulation and may possibly occur via upregulation of microglial Abeta phagocytosis (Yeh et al., 2016). Intraperitoneal (Montoliu-Gaya et al., 2018) or intraventricular (Qi et al., 2018) administration of a short clusterin-derived peptide can decrease Abeta deposition in mouse models, as well as ameliorate cognitive defects (Qi et al., 2018). This peptide corresponds to a D-amino acid version of one of clusterin’s predicted amphipathic helices (Bailey et al., 2001) and may play a role in recognizing Abeta (Wyatt et al., 2009) or in stimulating phagocytic activity, perhaps by upregulation of LRP2 (Qi et al., 2018) and thereby impacting clearance of Aβ across the blood–brain barrier.

In ALS patient tissues, immunoreactive clusterin is localized within cytoplasmic inclusions of TDP-43 in motor neurons, whereas in control tissues, clusterin staining is primarily found in the ER (Gregory et al., 2017). Further, in ALS mismatch cases – those patients with high TDP-43 burden but without cognitive deficits – clusterin expression was increased in gray matter neurons, compared to controls and ALS cases with cognitive decline. Additionally, glial clusterin expression was higher in cases of ALS with cognitive decline than in controls or mismatch cases (Gregory et al., 2020). Importantly, this effect could not be ascribed to general proteostatic differences, as the levels of the cytoplasmic chaperone HSPB8 were unchanged between controls and either disease case. These data suggest that the specific increase in neuronal expression of clusterin may be protective in an autologous fashion. It remains to be determined if elevations in clusterin are a consequence of a distinct disease progression that results in reduced cognitive decline, or instead represent a homeostatic means to provide neurological protection. It will also be important to assess whether similar proteostatic pathway changes are found in mismatch cases in other neurodegenerative diseases, such as AD, in which some diagnosed patients are known to have low levels of CSF Abeta, but no Abeta accumulation is visible in positron emission tomography (Mattsson et al., 2015).

Single nucleotide polymorphisms in clusterin have also been linked to early cognitive decline in PD (Gao et al., 2011; Sampedro et al., 2020). Clusterin has also been investigated as a potential therapeutic for PD. The preincubation of α-synuclein oligomers with clusterin prevented cell death and the production of reactive oxygen species, as well as TLR4 (toll-like receptor 4) activation (Hughes et al., 2019).

### Summary

It is clear that clusterin plays a variety of different roles throughout the brain (Wilson and Zoubeidi, 2017). While we do not yet fully understand the many mechanisms by which the clusterin chaperone operates to provide such a strong genetic link to AD, several common themes have emerged from research during the past two decades. With a client ratio of 1:10 for blocking Abeta amyloid formation (Yerbury et al., 2007), clusterin clearly represents a potent chaperone, and increasing evidence indicates that its extracellular chaperone action may directly impact the course of neurodegenerative disease. In addition, extracellular clusterin appears to act as a positive force in the endocytosis of (potentially toxic) aggregates and oligomers. Lastly, clusterin expression influences clearance of Abeta from the brain. Further research will be required to determine whether a specific mechanism represents a dominant phenotype, and whether other genes work together with clusterin to provide neuroprotection.

## Progranulin

Progranulin (gene name: *GRN*), a secreted, cysteine-rich glycoprotein, is highly expressed in cells of myeloid lineage such as macrophages and microglia; in epithelial cells; in a subset of neurons in the cortex, hippocampus, and cerebellum; and in motor neurons. Unlike other chaperones discussed in this review, progranulin functions as a personal chaperone for lysosomal proteins (reviewed in Bateman et al., 2018); however, it is secreted, suggesting extracellular action. Mammalian progranulin contains seven and a half granulin (GRN) domains, interspersed with variable length linkers. These GRN domains contain repeats of a 12-cysteine motif which can fold into tight beta-sheets linked by disulfide bonds, forming a “beads-on-a-string” arrangement. Progranulin can be cleaved into individual GRNs which can have functions independent of progranulin.

### Structure and Expression

The GRNs are referred to alphabetically by order of discovery, or numerically by order within progranulin, with “P” referring to the partial GRN in each case. From the amino-terminus, the order of the GRNs is P-G(1)-F(2)-B(3)-A(4)-C(5)-D(6)-E(7). Progranulin is secreted as a homodimer (Nguyen et al., 2013). Computational algorithms to determine protein disorder predict a high-low pattern where the more disordered GRN-G, GRN-B, GRN-C, and GRN-E are separated by the more ordered GRN-F, GRN-A, and GRN-D (Ghag et al., 2017). Further, recombinant GRN-B, while predicted to be disordered, forms a highly thermodynamically stable monomer, though when disulfide bonds are reduced, it becomes completely disordered (Ghag et al., 2017). This interspersing of order with disorder maybe be crucial to the chaperone activity of progranulin.

In the brain, expression of the progranulin gene is strong in microglia (Daniel et al., 2000; Baker et al., 2006; Ryan et al., 2009). *GRN* expression is also high in motor neurons in the spinal cord (Ryan et al., 2009). Progranulin is secreted in an activity-dependent manner at the synapse, with increasing neuronal activity correlating with increased progranulin levels within axons (Petoukhov et al., 2013). Thus, progranulin is appropriately expressed in extracellular locations where aggregating proteins and peptides are known to be released. At the transcriptional level, expression of progranulin is regulated by the master lysosomal biogenesis regulator transcription factor EB (TFEB) (reviewed in Kao et al., 2017), allowing for increased expression when increased degradative capacity is required.

Progranulin is found both in the lysosome and extracellular space. It reaches the lysosome either directly from the *trans*-Golgi, or after secretion and endocytosis via sortilin (Hu et al., 2010). In neurodegenerative diseases, progranulin and/or GRNs are detected within amyloid deposits (Gliebus et al., 2009; Gowrishankar et al., 2015; Mendsaikhan et al., 2019b), suggesting that progranulin may have additional extracellular roles in the formation of neurodegeneration-related protein aggregates.

### Function and Mechanism

Progranulin is known to exert a protective chaperone function on certain lysosomal enzymes [cathepsin D and beta-glucocerebrosidase (GCase)]. *In vitro*, progranulin added to recombinant cathepsin D protects this protein from high temperature-induced denaturation/degradation (Beel et al., 2017). Progranulin stabilizes the propeptide of cathepsin D, promoting autocatalysis at the active site (Butler et al., 2019). In the absence of progranulin, levels of cathepsin D (both pro and mature) accumulate, although enzyme activity decreases, a key indicator of lysosomal dysfunction (Götzl et al., 2018). The final 98 amino acids of progranulin, corresponding to GRN-E plus a linker sequence, are required for binding GCase, and also function to recruit non-canonically translocated HSP70 in a ternary interaction (Jian et al., 2016). We speculate that the intrinsic disorder predicted in both GRN-E and the C-terminal tail is important for recognizing multiple client proteins and for chaperone function.

A variety of *in vitro* and *in vivo* evidence supports a chaperone role for secreted GRNs. Recombinant GRN-B potently increases the fibrillation of Abeta *in vitro*, while reducing toxic oligomer formation (Bhopatkar et al., 2019). Abeta incubated under oligomerizing conditions with GRN-B at equimolar concentrations exhibits reduced caspase activation compared to Abeta incubated alone. However, GRN-B also seems to promote the formation of insoluble TDP-43 inclusions, exacerbating TDP-43 cytotoxicity (Bhopatkar et al., 2020); this is consistent with effects observed in *C*. *elegans* during GRN-B overexpression (Salazar et al., 2015). This aggregate promotion effect is increased following reduction of GRN-B; Bhopatkar et al. (2020) have speculated that the instability of reduced GRN-B can better disrupt proper TDP-43 folding, thus promoting its aggregation. Interestingly, known human mutations in GRN-B which disrupt the predicted beta-hairpin stack structure are linked to FTD, suggesting that the stacked structure is important for chaperone activity (van der Zee et al., 2007). Similar studies with GRN-C and TDP-43 show that GRN-C reduces thioflavin T-positive fibrillation of TDP-43 and promotes TDP-43 liquid-liquid phase separation *in vitro* (Bhopatkar et al., 2020). Taken together, these results suggest that certain individual secreted GRNs may function as sequestrase chaperones to remove aggregating proteins from solution and prevent the accumulation of small soluble toxic oligomers. The remaining GRNs and full-length progranulin remain to be mechanistically studied for *in vitro* chaperone activity.

### Disease Relevance and Therapeutic Potential

A variety of mutations in the progranulin gene are now known to result in FTD; these diminish levels of secreted progranulin either through reduced translation or via improper folding (Baker et al., 2006; Cruts et al., 2006; Chen et al., 2015). In FTD patients who lack *GRN* mutations (the more common form of FTD), CSF progranulin levels are still reduced as compared to healthy controls, suggesting that indirect changes in progranulin levels might contribute to these forms of FTD (Wilke et al., 2017). Loss of progranulin results in degeneration of the frontal and temporal lobes, causing dementia and finally death. Studies of rare individuals with two faulty copies of *GRN*, and of mouse progranulin knockout models, both support a severe impact of progranulin loss on lysosomal function; in humans, the complete loss of progranulin results in lysosomal storage disease (Ahmed et al., 2010; Smith et al., 2012).

In brain samples from patients with AD, immunoreactive progranulin colocalizes with Abeta deposits (Gowrishankar et al., 2015). Progranulin immunoreactivity is interspersed within most Abeta plaques in low pathology AD brains with fewer and smaller plaques (Mendsaikhan et al., 2019a), suggesting extracellular chaperone action. In a mouse model with mild amyloid formation, reduced progranulin levels increase amyloid deposition in the brain, while in the 5xFAD mouse model with high amyloid formation, overexpression of progranulin decreases Abeta plaque load (Minami et al., 2014). Genetic evidence also links progranulin to AD; the rs5848 polymorphism is linked to a 1.36-fold increased risk of late onset AD (Chen et al., 2015). This same polymorphism has been linked to increased risk of hippocampal sclerosis, as well as to increased CSF levels of tau (Fardo et al., 2017). As AD progresses, CSF levels of progranulin increase in the same time frame as neurodegeneration and neurofibrillary tangle formation occur (Suárez-Calvet et al., 2018).

Given the genetic risks associated with low progranulin levels in FTD and AD, increasing brain levels of progranulin via recombinant protein, viral delivery, or small molecule regulators, have all been proposed as potential disease treatments (reviewed in Gass et al., 2012). Determining the appropriate dosage will be critical, since adeno-associated virus- (AAV)-mediated overexpression of progranulin can cause hippocampal toxicity and neuronal degeneration via increased infiltration of T cells into the brain (Amado et al., 2019). Increasing progranulin expression can also induce ER stress in a variety of cell types (Li et al., 2015), likely due in part to the large number of disulfide bonds and complex folding. Nevertheless, a variety of studies are currently ongoing which involve therapeutic modulation of progranulin (recently reviewed in Cui et al., 2019). Of these, lentivirus-mediated overexpression of progranulin has likely been the most successful, and has been shown to reduce plaque burden and synapse loss in a mouse model of AD (Van Kampen and Kay, 2017). This same group had earlier shown that viral progranulin delivery into the substantia nigra protects dopaminergic neuronal health in a mouse model of PD (Van Kampen et al., 2014). In a null GRN mouse background, AAV-expressed progranulin rescues lysosomal function and reduces lipofuscinosis (Arrant et al., 2018). Oral administration of the disaccharide trehalose, an upregulator of autophagy, also increases progranulin expression in a haploinsufficient mouse model (Holler et al., 2016). Taken together, these data suggest that upregulation of progranulin expression, while potentially challenging to accomplish, may represent a promising treatment for FTD, AD, and PD.

### Summary

Modulating progranulin levels as a therapy in neurodegenerative disease shows great promise. However, achieving efficacious progranulin upregulation will require therapeutic discrimination between its various pro-growth, inflammatory, and chaperone activities. To better understand the role of progranulin as a general chaperone, *in vitro* assays of the effects of full-length progranulin will be required to complement the current studies of the individual domains on the aggregation and fibrillation of a variety of toxic proteins. Experiments defining the interactions between progranulin and/or individual GRNs with aggregating proteins are only now being attempted with mechanistic scrutiny (Bhopatkar et al., 2020).

## The BRICHOS Domain

The BRICHOS domain was originally found in, and named after, a set of chaperone proteins – Bri2, chondromodulin-1 and proSP-C – which demonstrate anti-amyloidogenic properties. Since its discovery in 2002, the BRICHOS domain has been found in 12 different protein families, with expression in various tissues. BRICHOS-domain proteins are ER membrane proteins and contain the BRICHOS domain in the C-terminal region; this domain is cleaved off by furin and other enzymes in the secretory pathway lumen and can then be secreted out of the cell. Interestingly, mutations in the genes encoding these proteins act as causative disease agents, for example Bri2 in dementias, chondromodulin-1 in cancer, and proSP-C in lung fibrosis. Cleaved products of Bri2 and proSP-C are also prone to form amyloid deposits. Abeta has been well studied as a client of the BRICHOS chaperone domain (reviewed in Willander et al., 2011; Buxbaum and Johansson, 2017).

### Structure and Localization

BRICHOS-domain containing proteins are generally ER-based type-II transmembrane proteins which contain a BRICHOS domain connected to the N-terminal transmembrane domain via a linker region. A 17-amino acid C-terminal luminal end is cleaved off by proteases, such as furin, forming the mature protein (Kim et al., 1999; Wickham et al., 2005). The luminal domain of Bri2 is further cleaved by ADAM10, thereby releasing the roughly 100-amino acid long BRICHOS domain into the secretory pathway (Martin et al., 2008). The crystal structure of recombinant human proSP-C BRICHOS domain shows beta sheets flanked by two alpha helices, with a tendency to form dimers and oligomers (Willander et al., 2012). All BRICHOS domains contain three conserved amino acids, two cysteines and one aspartic acid. The proteins also contain regions with the non−polar residues valine, isoleucine, phenylalanine and cysteine, which are prone to form beta-sheets with a tendency to aggregate (reviewed in Buxbaum and Johansson, 2017). While proSP-C (encoded by *SFTPC*) is expressed in the lungs, Bri2 (encoded by *ITM2B*) is expressed ubiquitously, and Bri3 (encoded by *ITM2C*) is brain-specific; both Bri2 and Bri3 are highly expressed in the hippocampus, cerebellum and cerebral cortex (Akiyama et al., 2004). In neuronal cell lines, Bri2 and Bri3 have been localized to the ER and Golgi complex, as well as within neurites (Martins et al., 2016). Localization of Bri2, but not its proteolytic cleavage product, to the plasma membrane appears to be regulated by glycosylation (Tsachaki et al., 2011).

### Function and Mechanism

Bri2 physically interacts with several proteins involved in membrane trafficking and the cytoskeleton (Martins et al., 2018). Several different BRICHOS-containing proteins were identified within the post-synaptic compartment in a mass spectrometry study and implicated in vesicle recycling, neurite growth and plasticity, neuronal differentiation, and signaling (Martins et al., 2018). In particular, Bri2 and Bri3 have been found to be physically complexed with, and be phosphorylated by, PP1, which regulates its functions in neurite growth and neuronal differentiation, as well as its proteolytic processing (Martins et al., 2016, 2017). Bri2 and Bri3 bind to APP during biosynthesis, shielding it from the secretases involved in APP cleavage, and reducing the production of the aggregation-prone peptide products Abeta40 and Abeta42 (Matsuda et al., 2005, 2011). These studies indicate a role for Bri2 and Bri3 in the functional regulation of APP processing under normal conditions.

Different *in vitro* studies point to a variety of possible mechanisms proposed to explain how recombinant human BRICHOS might modulate Abeta oligomerization, namely via blocking secondary nucleation and/or by blocking fibril formation and elongation. *In vitro*, the various recombinant BRICHOS domains of Bri2, Bri3 and proSP-C have all been shown to reduce toxic Abeta oligomerization and fibrillation (Biverstal et al., 2015; Dolfe et al., 2016). The assembly state of the BRICHOS domain appears to dictate its specific effect on amyloid oligomerization and fibrillation. Recombinant BRICHOS monomers adopt different quaternary structures; they also form dimers, which act as the subunits for oligomerization (Chen et al., 2017). The monomeric and dimeric states have been shown to be more potent at reducing Abeta fibril formation as compared to oligomeric states (Cohen et al., 2015; Chen et al., 2017). Dimers also appear to be more potent than monomers in reducing Abeta fibril elongation and secondary nucleation by binding to Abeta fibrils (Cohen et al., 2015; Chen et al., 2017).

The BRICHOS domain mutations R221E and S95R, which promote a stable monomeric state, were found to significantly reduce secondary nucleation and to delay fibrillation *in vitro*, respectively, as compared to mutant oligomeric states (Biverstal et al., 2015; Chen G. et al., 2020). Immunogold electron microscopy showed that formation of Abeta fibrils *in vitro* was drastically delayed in the presence of recombinant Bri2 and proSP-C BRICHOS domains, likely due to the fact that BRICHOS domain binds to Abeta fibrils, shielding it from further surface nucleation (Willander et al., 2012). More recently, cryo-EM studies showed that the process of Abeta fibrillation consisted of both formation of free floating protofibrils as well as fibrils with surface nucleation; in the presence of the BRICHOS domain, a higher number of free-floating Abeta protofibrils were found, while in its absence, secondary nucleation was favored (Tornquist et al., 2020). This supports the idea that the possible mechanism of action is by reducing secondary nucleation rather than prevention of *de novo* fibril formation. Interestingly, BRICHOS domain oligomers are also able to reduce non-fibrillar aggregation of Abeta (Chen et al., 2017). Taken together, these biochemical studies strongly support a role for the BRICHOS domain to act as an anti-aggregant for Abeta, and additionally identify monomeric variants that could be tested in cell culture and animal models to provide effective neuroprotection against Abeta toxicity.

### Disease Relevance and Therapeutic Applications

Genetic evidence supports the association of BRICHOS domain-containing proteins with neurodegenerative disease. Read-through mutations in Bri2, leading to the expression and cleavage of an additional 11 amino acids in the C-terminal peptide product, result in the formation of amyloid protein deposits in the brain, and are responsible for the familial British and Danish dementias (FBD and FDD, respectively) (Rostagno et al., 2005). Bri2 and Bri3 BRICHOS domains have been found to be colocalized with amyloid plaques in AD patients (Del Campo et al., 2014; Dolfe et al., 2018). Bri2 and Bri3 were also shown to be able to bind Abeta and/or APP in the CA1 region of the hippocampus in transgenic APP mice (Dolfe et al., 2018). However, while levels of Bri2 levels were increased in AD patient brains, levels of Bri3 were reduced (Dolfe et al., 2018). In cell culture, Bri2, but not Bri3, has been detected in the medium of overexpressing cell lines (Dolfe et al., 2018). The dissimilarities between Bri2 and Bri3 levels and localization support differences in mechanism of action between the two chaperones. While the Bri2 BRICHOS-domain probably interacts with Abeta extracellularly and/or upon reuptake, it is likely that Bri3 BRICHOS-domain binds Abeta only within the secretory pathway.

*In vivo* and *ex vivo* studies highlight the importance of BRICHOS-containing proteins in combating Abeta toxicity. Although *in vitro*, recombinant BRICHOS dimers exhibit higher activity against Abeta fibrillation than monomers, physiologically, it is the BRICHOS monomers rather than dimers that reduce Abeta-induced damage to neuronal networks in mouse hippocampal slices treated with Abeta. However, these studies involve the use of BRICHOS domain protein at the relatively high chaperone: client molar ratio of 1:1 (Chen et al., 2017). Recombinant Bri2 BRICHOS domain and its monomeric variant, R221E, were also able to reduce cytotoxic effects induced by exposure of hippocampal slices to Abeta42 monomers, and also partially to preformed Abeta fibrils (Poska et al., 2016; Chen G. et al., 2020). In a *Drosophila* model of AD, coexpression of Bri2 with Abeta42 reduced Abeta aggregation in adult brains as well as Abeta-induced retinal degeneration; overexpressed Bri2 also rescued lifespan and motor defects (Hermansson et al., 2014). In the presence of Bri2, Abeta42 became diffusely colocalized with Bri2 in the mushroom bodies, the seat of cognition and learning in the adult *Drosophila* brain, instead of exhibiting the punctate morphology observed in the absence of Bri2 (Poska et al., 2016), providing evidence that Bri2 can prevent Abeta42 deposition *in vivo*. In a mass spectrometry screen, the ubiquitin ligase NRBP1 was recently found to be a substrate receptor for Bri2 and Bri3, recruiting these proteins during ERAD for ubiquitination by the Cullin-RING ligase complex and thereby targeting them for proteasomal degradation (Yasukawa et al., 2020). Transgenic mice expressing fused Bri2-Abeta40/42 exhibited delayed formation of Abeta plaques, and were devoid of cognitive or behavioral decline, supporting the sequestering activity of Bri2 as a likely mechanism to prevent the spread of toxic oligomers and reduce neuronal death (Kim J. et al., 2013). Reduced cognitive decline in Bri2-Abeta40/42 expressing transgenic mice compared to controls suggests that this specific mechanism normally serves to prevent or delay processes that much later result in the development of AD (Kim J. et al., 2013). A similar effect has not been identified in AAV-mediated expression of Bri-Abeta40/42 in rats, which developed pathological symptoms of AD (Lawlor et al., 2007). Efforts to increase Bri2 and Bri3 expression could prove to be therapeutic in AD by regulating events from Abeta processing to fibril formation.

### Summary

The data obtained to date indicate that BRICHOS domain-containing proteins–while themselves susceptible to amyloid formation–can effectively combat Abeta-mediated cytotoxicity at various stages: by preventing APP cleavage and Abeta toxic peptide production, as well as by interacting with Abeta to reduce fibrillation both within the secretory pathway and the extracellular milieu.

## 7B2 and ProSAAS

While all other secreted chaperones discussed in this review are expressed ubiquitously in the body, there are two small chaperone proteins, 7B2 and proSAAS, whose expression is mainly restricted to cells containing a regulated secretory pathway, namely neurons, neuroendocrine, and endocrine cells. Both proteins are similar in size, around 250 amino acids, contain functionally similar core segments, and are cleaved at least once by the proprotein convertase furin, releasing about-20 kDa N-terminal domains with chaperone functionality. However, 7B2 and proSAAS have no amino acid homology. Interestingly, 7B2 is an ancient protein, found in organisms as primitive as flatworms and rotifers (see PFAM PF05281) while the proSAAS protein is much more recent, first appearing only in vertebrates (see PFAM PF07259).

### Structure and Localization

The neuroendocrine chaperone 7B2 (gene name *SCG5*) was first identified in neuroendocrine tissues by direct peptide sequencing over 28 years ago (Hsi et al., 1982); it is predominantly expressed in pituitary, all areas of the brain, pancreas, and adrenal (Iguchi et al., 1984, 1985). Within the cell, 7B2 is concentrated within regulated secretory granules, from which it is released following stimulation (Iguchi et al., 1987b); see also review (Mbikay et al., 2001). CSF concentrations approximate 3 ng/ml or 0.14 nM (Iguchi et al., 1987a) and decline with age (Natori et al., 1987). In a specific substrain of mice, loss of 7B2 results in a lethal phenotype between 5 and 8 weeks of age owing to hypersecretion of ACTH from the pituitary (Westphal et al., 1999; Laurent et al., 2002). These data indicate a possible role for 7B2 in peptide hormone storage. One early study indicates that the C-terminal peptide can depolarize vasopressin- and oxytocin-containing neurons in hypothalamic explants (Senatorov et al., 1993). However, no other studies have shown neuropeptide-like actions for 7B2, and no receptors for 7B2-derived peptides have been identified.

Full-length 27 kDa 7B2 is cleaved by Golgi-resident furin, releasing a 21 kDa product (Ayoubi et al., 1990). Both 27 kDa and 21 kDa 7B2 contain a central IDR, as indicated both by the PONDR prediction algorithm and a lack of secondary structure detected by circular dichroism, with the 27 kDa form being more compact than 21 kDa form (Dasgupta et al., 2012).

ProSAAS (gene name *PCSK1N*) is an abundantly expressed brain protein discovered 20 years ago using mass spectrometric techniques applied to brain peptide extracts (Fricker et al., 2000). Like 7B2, it is predominantly expressed within the brain as well as in endocrine and neuroendocrine tissues (Lanoue and Day, 2001; Sayah et al., 2001; Morgan et al., 2005), where, like 7B2, it is stored within secretory granules (Wardman et al., 2011; Wardman and Fricker, 2014). While the 7B2-encoding gene contains a heat shock-responsive element (Martens, 1988; Mbikay et al., 2001), the mouse or human proSAAS-encoding genes do not. Interestingly, heat shock does increase the quantity of cellular proSAAS in cell culture (Shakya et al., 2020). Expression of the *Pcsk1n* gene in differentiating neural tube neurons was observed in developing rat embryos as early as 12 days of gestation, while proSAAS processing begins in mid-gestation (Morgan et al., 2005). Due to the lack of an adequately sensitive radioimmunoassay, the concentration of proSAAS in CSF and in plasma is not yet known, but brain concentrations have been estimated to be between 10 and 500 nM depending on region (Jarvela et al., 2016).

Within the secretory pathway, basic residue pairs within the amino- and carboxy-terminal portions of 27 kDa proSAAS are cleaved by proprotein convertases and carboxypeptidase E to produce various secreted peptide products (Fricker et al., 2000; Mzhavia et al., 2001, 2002; Sayah et al., 2001; Wardman et al., 2011), and specific proSAAS-derived peptides are thought to have biological functions (Hatcher et al., 2008). The 21 kDa N-terminal domain of proSAAS, separated from the carboxy-terminal domain by a furin consensus sequence, harbors an internal predicted coiled coil region as well as a predicted IDR (Kudo et al., 2009), through which it potentially interacts with client proteins.

### Function and Mechanism

Thirteen years following its discovery, 7B2 was identified as an anti-aggregant chaperone for prohormone convertase 2 (proPC2) (Zhu and Lindberg, 1995; Lee and Lindberg, 2008), functioning through an evolutionarily conserved PPNPCP motif within a 36-residue region in the middle of the protein (Zhu et al., 1996; Muller et al., 1999). The chaperone activity of this anti-aggregant region is reminiscent of the α-crystallin-like domain within soluble sHSPs (see below), although the two types of proteins bear no sequence similarity. Like sHsps, 7B2 demonstrates tendencies to both dimerize and to exhibit concentration-dependent polydispersity (Dasgupta et al., 2012); we speculate that as in sHsps, the presence of IDRs as well as the formation of oligomers may be important for binding aggregation-prone proteins such as proPC2 (Lee and Lindberg, 2008), insulin-like growth factor (Chaudhuri et al., 1995), and islet amyloid polypeptide (Peinado et al., 2013).

Like 7B2, proSAAS is also capable of reducing the fibrillation of aggregating proteins. To date known proSAAS clients include Abeta (Hoshino et al., 2014); α-synuclein (Jarvela et al., 2016); and islet amyloid polypeptide (Peinado et al., 2013). Exciting new results indicate that cytoplasmic expression of proSAAS results in the formation of phase-separated proSAAS spheres which are able to trap the aggregating protein TDP-43^214–414^ within their cores (Peinado et al., 2020). Further structure-function analysis should permit us to determine the self-associating domains of proSAAS as well as the residues lining the sphere interior, which clearly favor aggregate binding.

### Disease Relevance and Therapeutic Applications

Evidence of extracellular action for 7B2 is its colocalization with Abeta plaques; immunoreactive 7B2 is also found in Lewy bodies in PD patient samples (Helwig et al., 2013). While levels of 7B2 in the CSF were shown to decrease during normal aging (Natori et al., 1987), three studies reported increased CSF 7B2 levels in FTD (Mattsson et al., 2008) and/or ALS patients (Ranganathan et al., 2005; Jahn et al., 2011). However, among AD patients, contradicting studies have reported either a slight increase (Winsky-Sommerer et al., 2003) or no change (Iguchi et al., 1987a) in 7B2 levels. APP transgenic mouse brains also do not show alterations in 7B2 levels, indicating that 7B2 is not upregulated during the course of disease (Jarvela et al., 2018). Majerova et al. (2017) showed increased levels of CSF 7B2 in proteomic studies of a tauopathy transgenic rat model of AD. While no genetic evidence directly implicates 7B2 in disease processes, the potent chaperone action of 7B2, and the presence of 7B2 in a variety of extracellular protein deposits supports the idea that brain 7B2 levels may be relevant to proteostatic processes in neurodegeneration.

Immunofluorescence studies have similarly shown that proSAAS co-localizes with aggregated proteins involved in neurodegenerative disease, namely tau tangles in dementia (Kikuchi et al., 2003); Abeta plaques in AD (Hoshino et al., 2014); and Lewy bodies in PD (Helwig et al., 2013). Seven independent proteomics studies have shown that the level of proSAAS in CSF taken from AD and/or FTD patients is reduced as compared to controls, suggesting possible cellular retention within the brain (Davidsson et al., 2002; Abdi et al., 2006; Finehout et al., 2007; Jahn et al., 2011; Choi et al., 2013; Holtta et al., 2015; Spellman et al., 2015; reviewed in Pedrero-Prieto et al., 2020). Two very recent CSF studies support this reduction (Rotunno et al., 2020; Van Steenoven et al., 2020).

The idea that proSAAS plays a role in neurodegenerative proteostasis is further supported by human transcriptomics studies, which indicate increased proSAAS expression during AD progression (Mathys et al., 2019). Increased levels of proSAAS have also been found in brain tissues of patients with CAA (Inoue et al., 2017), as well as in models of neurodegenerative diseases including horses (McGorum et al., 2016) and rats (Chatterji et al., 2014). Lastly, recent data from our laboratory indicate that cellular proSAAS levels are upregulated following ER and even heat stress (Shakya et al., 2020); interestingly, in parallel experiments, similar upregulation was not observed for 7B2. Lastly, in an experiment to determine which endogenous CSF proteins bind to the amyloid fold, proSAAS was identified (Juhl et al., 2019). Collectively, these data strongly support the involvement of proSAAS in proteostatic mechanisms of neurodegenerative disease.

Biochemical studies indicate possible similar mechanisms of action for 7B2 and proSAAS with respect to their ability to block aggregative processes in neurodegeneration. For example, *in vitro* fibrillation studies demonstrate that both chaperones potently reduce the oligomerization of aggregation-prone proteins (Helwig et al., 2013; Hoshino et al., 2014; Jarvela et al., 2016). Structure-function studies have revealed that for both proteins, a conserved internal domain of about 100 residues is responsible for anti-fibrillation chaperone activity (Helwig et al., 2013; Jarvela et al., 2016). Both proteins act at sub-stoichiometric client ratios; while both proSAAS and 7B2 are able to reduce the fibrillation of Abeta at a 1:10 chaperone: client molar ratio, proSAAS is able to efficiently diminish α-synuclein fibrillation at a molar ratio of 1:70. While both chaperones reduce Abeta and α-synuclein fibrillation, neither is able to disaggregate preformed fibrils, and the addition of ATP and/or HSP70 has no effect on their activity (Helwig et al., 2013; Jarvela et al., 2016). These results support the idea that these chaperones act alone rather than in concert with other chaperones or disaggregases. How these two chaperones are able to become trapped within aggregates is unclear, but if lessons from small cytoplasmic Hsps apply, then perhaps initial anti-aggregant activity is transformed into a sequestrase function as client levels progressively overwhelm chaperone levels (Mogk et al., 2019).

Limited studies in cell and animal models also support the idea of extracellular anti-aggregant action for proSAAS and 7B2. Both chaperones are cytoprotective in cell culture as well as in rodent models of AD and PD (Helwig et al., 2013; Hoshino et al., 2014; Jarvela et al., 2016). Application of recombinant 21 kDa proSAAS was found to reduce cytotoxicity in α-synuclein-expressing SH-SY5Y cells and in Abeta oligomer-treated Neuro2A cells, indicating effective extracellular chaperone function against these two aggregating proteins (Hoshino et al., 2014; Jarvela et al., 2016). Lentiviral expression of proSAAS increased the number of tyrosine hydroxylase-positive cells in rat primary nigral cell cultures expressing AAV-encoded α-synuclein (Jarvela et al., 2016). Similarly, external application of recombinant 21 kDa 7B2, as well as AAV-mediated overexpression of intact 7B2 in Neuro2A cells, were both cytoprotective against toxic Abeta oligomers (Helwig et al., 2013).

Paradoxically, APP model mice lacking 7B2 expression (by crossing with 7B2 knockout mice) exhibit a reduction rather than an increase in Abeta plaques (Jarvela et al., 2018). 7B2 null APP model mice also do not exhibit alterations in soluble Abeta, cognition, or memory compared to similar mice expressing 7B2 (Jarvela et al., 2018). These results are reminiscent of similar paradoxical results obtained in various crosses of clusterin knockout mice with APP model mice (DeMattos et al., 2002; Wojtas et al., 2017; Oh et al., 2019) which were attributed to the dominance of clearance effects rather than aggregate formation (Wojtas et al., 2017). We speculate that for both clusterin and 7B2, chaperone loss may directly result in lower extracellular aggregate sequestration through some as-yet undefined mechanism. However, as with clusterin, it is also possible that the loss of 7B2 impacts the expression of other genes, which indirectly causes the observed reduction in plaques. Whether the expression of either proSAAS or 7B2 impacts brain Abeta clearance is not yet known. Similar studies in proSAAS knockout mice (Morgan et al., 2010) have not yet been performed.

### Summary

Since proSAAS and 7B2 are secreted from neurons, are associated with protein deposits extracellularly, and (in the case of proSAAS), exhibit increased brain expression during the development of neurodegenerative disease, it is feasible to speculate that these chaperones act extracellularly to perform a protective proteostatic function. Thus, overexpression of these chaperones represents a potential approach for slowing the progression of AD and PD, and indeed our studies using a rat model of PD support the idea that manipulation of brain proSAAS levels is beneficial to disease outcome (Lindberg et al., 2018). Additional *in vivo* studies to decipher the physiological mechanisms involved in cytoprotection are needed to identify the precise biochemical contribution of these chaperones in disease processes. Similarly, additional *in vitro* structure-function experiments will shed light on the precise regions within each protein that contribute to anti-aggregant function.

## Small Heat Shock Proteins (sHsps)

The sHsp family of chaperones has been frequently reviewed within recent years, even specifically within the context of neurodegeneration (Kourtis and Tavernarakis, 2018; Muranova et al., 2019; Webster et al., 2019; Vendredy et al., 2020). These reviews have amply covered the clearly protective roles of intracellular sHSPs. For the purpose of this review, we will focus on studies relating to secreted sHsps in the context of neurodegenerative disease. Studies implicating extracellular mechanisms of sHsps in neurodegenerative disease fall into the following three categories: disease-associated analyses of biological fluids, such as CSF and plasma; demonstration of immunohistochemical association with extracellular protein aggregates; and direct cellular secretion experiments.

### Structure and Localization

All ten members of this family of chaperones (HspBs 1–10) are small proteins of less than 45 kDa and all possess a conserved α-crystallin domain (“ACD”) which is both required for chaperone action and responsible for the self-association phenomenon that creates molecular mass polydispersity. This domain is almost always flanked by two other domains; the N-terminal domain is about 50 residues, while the C-terminal domain may be quite short in some family members.

sHSPs are expressed in every tissue, but three family members are especially abundant in brain: HspB1, HspB5 and HspB8; HspB2, HspB3, HspB6, and HspB7 are also detected in brain but at much lower levels (Quraishe et al., 2008; Kirbach and Golenhofen, 2011). Interestingly, these abundant sHsps are predominantly expressed by non-neuronal cells such as glia, rather than by neurons (reviewed in Golenhofen and Bartelt-Kirbach, 2016), where they are found associated with intracellular aggregates in various tauopathies.

Within the cell sHSPs are predominantly located within the cytoplasm but may under certain circumstances be released from cells through various unconventional means that include exosomal and/or endolysosomal secretion, other mechanisms such as tunneling nanotubes, and even direct secretion (recently reviewed in Reddy et al., 2018; Webster et al., 2019). Astrocytes are able to release HspB1 via exosomes (Nafar et al., 2016), and retinal pigment epithelium cells also use exosomes to secrete HspB5 (Sreekumar et al., 2010); indeed, the latter chaperone may be required for exosome synthesis (Gangalum et al., 2016). In contrast, unconventional secretion of this chaperone from COS cells requires the autophagic pathway and is controlled by phosphorylation (D’Agostino et al., 2019). A dynamic relationship between sHsp secretion and extracellular proteostasis has not yet been established.

### Function and Mechanism

sHsps are important ATP-independent holdase chaperones that interact with monomers of aggregation-prone proteins to stabilize them in preparation for refolding or disposal. Most sHsps are found in homo-oligomers of 10–20 subunits, composed of dimer subunits interacting via the ACD (Kim et al., 1998). Through partial unfolding, sometimes in response to environmental stressors (Kirbach and Golenhofen, 2011; Alderson et al., 2020), they are able to interact with a variety of client proteins (reviewed in Webster et al., 2019). Supportive of an extracellular role in proteostasis, HspB5, also known as alpha crystallin, αBC, and CRYAB, has long been known as a potent anti-aggregant *in vitro* against a variety of fibrillating proteins; these studies span the last two decades [recently reviewed in Boelens (2020); see also Selig et al. (2020) and Bendifallah et al. (2020) for recent results concerning Abeta and synuclein, respectively].

Considerable evidence implicates sHsp family members in extracellular proteostasis. sHsp chaperones are frequently found associated with both intracellular as well as extracellular protein deposits (reviewed in Hilton et al., 2013; Reddy et al., 2018). Phosphorylation induces structural changes resulting in oligomer dissociation, which can be associated with reduced chaperone capacity (D’Agostino and Diano, 2010). The HspB1 chaperone (also known as Hsp27 in humans and Hsp25 in rodents) is a stress-responsive chaperone which both facilitates folding and acts as an antioxidant; the secretion of this chaperone under various cellular conditions – the majority of which are cancer-related – has been nicely summarized in Reddy et al. (2018). A large number of studies have demonstrated the presence of HspB5 within extracellular brain aggregates, supporting its secretion during proteostatic failure (recently comprehensively reviewed in Muranova et al., 2019; Webster et al., 2019; Vendredy et al., 2020). Extracellular chaperone action may occur through facilitation of the sequestration of Abeta-related species rather than by refolding attempts (Ojha et al., 2011). This phenomenon illustrates the apparent paradox of chaperone trapping within insoluble aggregates. Intracellularly, during stress when proteins become unstable, sHsps form an outer shell composed of dimer subunits that sequester early unfolded intermediates of these proteins in order to preserve their partially folded structure, thus preventing their interaction with one another (Mogk et al., 2019). Intracellular sHsps commonly require ATP-dependent chaperones to resolubilize unfolded “held” proteins; since these same ATP-dependent chaperones are apparently not secreted in sufficient quantities, secreted sHsps may operate mainly to assist extracellular sequestration events rather than assisting unfolding. The exact mechanism for extracellular sequestration is as yet unclear but could be similar to intracellular sequestration. However, client: chaperone ratios likely differ inside and outside the cell, which could impact chaperone shell formation and subsequent core sequestration. A high client: chaperone ratio may underlie chaperone trapping within protein deposits, whereas a low ratio might result in trapping of toxic oligomers inside the chaperone shell to prevent further aggregation.

In non-neuronal systems, secreted sHSPs appear to exert other extracellular roles, for example as signaling peptides or as peptides involved in immunity and inflammation (Arac et al., 2011; discussed in Reddy et al., 2018); whether this also occurs in brain, and how these other roles might interact with chaperone functions has not yet been established.

### Disease Relevance and Therapeutic Applications

Evidence of the involvement of sHSPs in neurodegenerative disease comes from studies of human mutations; for example, mutations in the three family members most abundant in brain, HspB1, HspB3, and HspB8, have been implicated in certain forms of Charcot-Marie-Tooth neuropathy and/or distal hereditary motor neuropathy (Muranova et al., 2019; Vendredy et al., 2020). Renkawek et al. (1999) found increased expression of HspB1 in PD; interestingly, this same group showed that HspB8, also known as Hsp22, is upregulated in brains from AD model mice (Wei, 2020). This chaperone colocalizes with extracellular amyloid deposits found in CAA, a common comorbid condition in AD (Wilhelmus et al., 2009). A recent meta-analysis of protein quality control pathways in the AD brain clearly shows upregulation of sHSPs (Koopman and Rüdiger, 2020). Multiple immunohistochemical studies have shown that sHsps are present within extracellular amyloid plaques (Wilhelmus et al., 2006; Ojha et al., 2011; see Reddy et al., 2018 for review). Coupled with multiple reports of upregulation of sHsp expression in AD and other neurodegenerative diseases (discussed further in Webster et al., 2019) these findings support the idea that sHsps may operate extracellularly within the brain to reduce Abeta toxicity in AD.

With regard to other neurodegenerative diseases, elevated serum levels of HspB1 have been reported during attacks in multiple sclerosis (Ce et al., 2011). However, in a large proteomics meta-analysis of Alzheimer’s CSF biomarkers, no sHSPs were identified as differentially expressed in any of the over 40 collated studies (Pedrero-Prieto et al., 2020) indicating that AD progression does not involve alterations in the secretion of sHsps into the CSF. The extracellular (blood) presence of HspB5 has been demonstrated by inference in the form of autoantibodies found in the sera of AD and PD patients (Papuc et al., 2016).

Many studies have shown that overexpression of HspB5 is neuroprotective in a variety of cell and animal model systems (reviewed in Kourtis and Tavernarakis, 2018; Muranova et al., 2019; Webster et al., 2019; Vendredy et al., 2020).

With regard to therapeutic applications, the large number of interacting client proteins renders the notion of specific drug-induced enhancement of a specific aggregating target problematic. However, Rothbard et al. (2019) have recently demonstrated that administration of HspB5 was therapeutic in animal models of multiple sclerosis, retinal and cardiac ischemia, and stroke.

### Summary

The sHsps are a group of small chaperones with no energetic requirements for client binding which efficiently bind to a large number of aggregated proteins involved in neurodegenerative disease. While they are clearly secreted (mostly from glia) under certain circumstances, we are only now beginning to understand the non-canonical secretion mechanisms which might allow these abundant cellular proteins to assist in extracellular proteostasis. We clearly also need a more complete understanding of the biochemical mechanisms underlying the extracellular sequestration of aggregating proteins by sHSPs.

## General Discussion

In this review, we have attempted to summarize the available information on select secreted chaperones associated with neurodegenerative disease, using biochemical and genetic evidence to focus on those proteins with the strongest evidence for both proteostatic as well as extracellular actions in neurodegeneration.

### Common Mechanisms of Action?

A common theme in many of the chaperones discussed above is the presence of a specific domain functionally similar to the α-crystallin domain which is required for chaperone activity. In sHsps, this is the ACD itself; for clusterin, this may involve residues 286–343, which exhibit 25% similarity with a canonical ACD (Wilson and Easterbrook-Smith, 2000). In BRICHOS domain-containing proteins, and in 7B2 and proSAAS, an interior segment of about 100 residues, with no sequence similarity to α-crystallin, is required for chaperone activity. A similar functional segment has not yet been identified in progranulin. In sHsps, these same sequences also function to promote self-association (Kim et al., 1998; Wyatt et al., 2009). Clusterin, 7B2 and proSAAS also form polydisperse assemblies; we speculate that similarly to sHsps, polydispersity results in the exposure of a range of different intrinsically disordered surfaces, permitting these chaperones to bind diverse clients.

**FIGURE 1:**
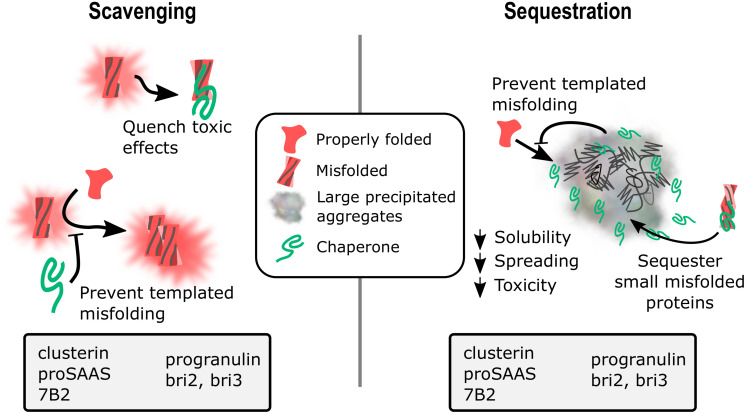
Potential mechanisms for extracellular chaperone action in neurodegenerative disease: Scavenging and sequestration. A schematic diagram showing two proposed mechanisms of action for extracellular chaperone activity on misfolded and aggregating substrates. (1) Scavenging. Chaperones can bind soluble monomers and low molecular weight oligomers to prevent toxic effects from the misfolded protein as well as block the template misfolding/aggregation of natively folded monomers. Chaperones with data showing the ability to block aggregation of misfolded proteins are listed in the gray box. (2) Sequestration. Chaperones can interact with soluble misfolded proteins and drive their recruitment into insoluble aggregates, reducing toxic species solubility, spreading ability, and toxicity. Chaperones can also serve as a buffer to block templated misfolding of natively folded proteins, and may also interact with insoluble aggregates. Chaperones with data showing colocalization with amyloid plaques, neurofibrillary tangles, Lewy bodies, and/or intracellular inclusions are listed in the gray box.

None of the chaperones discussed in this review work together with other ATP-dependent chaperones to refold proteins, but instead appear to act as holdases to bind unstable protein monomers and small oligomers, initially to prevent aggregation/fibrillation, with evidence suggesting both extracellular and intracellular scavenging action (Figure 1, left panel). Presumably, when overwhelmed with client, these chaperones act as sequestrases to bind and sequester toxic oligomers, potentially leading to the formation of insoluble protein deposits such as extracellular plaques and intracellular Lewy bodies (Figure 1, right panel). These chaperones, like their client counterparts, contain IDRs of varying degrees that essentially enable the holdase and sequestrase activities, and allows them to form functional multimers that can bind to toxic protein species, preventing cellular damage. Support for this mechanism has been presented for clusterin, BRICHOS proteins, and cytoplasmic sHsps (Rohne et al., 2016; Chen et al., 2017; Mogk et al., 2019) and recent work shows similar properties for GRN-C (Bhopatkar et al., 2020) and for proSAAS (Peinado et al., 2020). In addition, the finding of reduced plaque number in AD model mice lacking 7B2 expression (Jarvela et al., 2018) or clusterin (DeMattos et al., 2002; Wojtas et al., 2017) suggests possible roles for 7B2 and clusterin in sequestration events (Figure 1, right panel).

**FIGURE 2:**
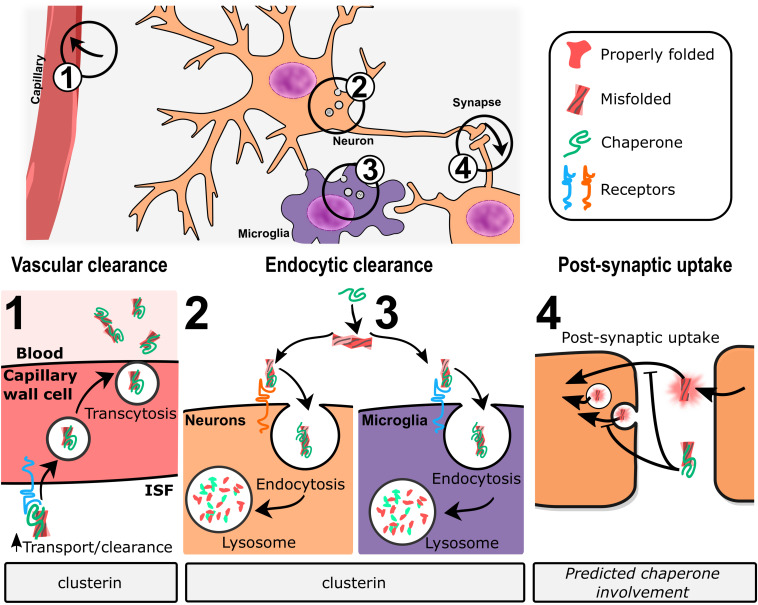
Potential mechanisms for extracellular chaperone action in neurodegenerative disease: Clearance and transmission. A schematic diagram of proposed mechanisms of action of extracellular chaperones activity on transmission and clearance of misfolded and aggregating substrates. (1) Vascular clearance. Chaperone–client complexes are recognized by cell surface receptors on the capillary endothelium for transcytosis from the interstitial fluid (ISF) to the blood for removal. Chaperones that promote clearance across the blood–brain barrier are listed in the gray box. Endocytic clearance by (2) neurons or (3) microglia. Chaperone–client complexes interact with cell surface receptors on to promote the endocytosis and lysosomal degradation of chaperone–client complexes. Chaperones that show increased endocytosis and degradation of proteopathic seeds are listed in the gray box. (4) Post-synaptic uptake. Chaperones bind to secreted proteopathic seeds at the synapse to prevent their uptake (through either direct membrane penetration or endocytosis) by the post-synaptic neuron to reduce cell-to-cell transmission of disease pathology. (Note that no chaperone has yet been directly implicated in this pathway).

Possible mechanisms of chaperone action downstream of misfolded protein sequestration have been identified for clusterin and could serve as plausible pathways for other chaperones. Clusterin mediates the clearance from the plasma (via the liver and kidney) and CSF (via transcytosis across the blood–brain barrier (BBB) of a variety of client proteins (Figure 2, panel 1). Clusterin also promotes endocytosis and degradation via non-professional phagocytic cells, through binding cell surface HSPGs (Itakura et al., 2020) (Figure 2, panel 2) and via microglial cells by binding the surface receptor TREM2 (Triggering Receptor Expressed on Myeloid Cells 2) (Yeh et al., 2016) (Figure 2, panel 3). Similar membrane receptors in brain cells promote extracellular clearance of client-chaperone complexes. These processes would essentially prevent the likely spread of toxic oligomeric species across the blood–brain barrier (Figure 2, panel 1) and between neurons (Figure 2, panel 4). Of note, transsynaptic transmission of misfolded proteins is increasingly recognized as a major source of spread of pathogenesis across brain regions (Peng et al., 2020).

### Remaining Questions

Much additional *in vivo* work is required to document the protective role of each of these chaperones *in vivo*. For example, while there is evidence that intravenous administration of recombinant clusterin in mice can reduce insoluble Abeta levels and diminish hippocampal neuronal loss, clusterin-deficient mice models expressing human APP/presenilin-1 have decreased rather than increased numbers of hippocampal Abeta plaques; similar results were found with 7B2-deficient mice. While these paradoxical results may be partially explained by predominant effects on Abeta clearance, no direct evidence supports this idea; and overexpression of other chaperones, including sHsps, has been shown to be beneficial in animal models of neurodegenerative disease (reviewed in Kourtis and Tavernarakis, 2018; Webster et al., 2019). The cleaved BRICHOS domain has been shown to be anti-amyloidogenic *in vitro*, and cytoprotective *ex vivo* in hippocampal slices and *in vivo* in *Drosophila* models of AD and likely also in transgenic mice expressing fused Bri2-Abeta40/42. However, *in vivo* data from deficient or transgenic mice crossed with mice modeling neurodegenerative disease are lacking for many of the other chaperones discussed here, including proSAAS, BRICHOS domain-containing proteins and progranulin.

Recent studies have identified the transmission of pathological protein aggregates between cells as an important mechanism underlying the progression of a variety of neurodegenerative diseases (reviewed in Peng et al., 2020). Thus, the role of the chaperones discussed here should be examined using experimental paradigms that can discern cell-to-cell transmission *in vivo* (Figure 2, panel 4), which have now been published for α-synuclein, Abeta, tau, and TDP-43 (reviewed in Peng et al., 2020). Experiments involving supplementation or depletion of individual chaperones using these spread paradigms should provide new mechanistic insights of extracellular chaperone function.

Another interesting question is the contribution of secreted chaperones to neuronal health under normal conditions. The lack of animal models for many of these chaperones represents a gap in our understanding of their mechanisms of action under healthy conditions. Alternative mechanisms of how these chaperones protect against disease can be obtained by elucidating their normal chaperone functions *in vivo*. Bri2 and Bri3, for example, are known to suppress the cleavage of APP in order to prevent formation of toxic peptides, potentially representing an effective mechanism to delay AD pathology; this may or may not occur during the normal lifespan. For progranulin, its many other bioactivities (pro-growth and anti-inflammatory) are not clearly related to its chaperone roles, which provides an additional complication; however, attempts should be made to tease out its specific chaperone contributions to neuroprotection.

Clear evidence of cytoprotective activity in cell culture and animal disease models warrants the pursuit of many of these chaperones as therapeutics, and indeed several of the chaperones discussed are already being exploited as pharmacologic agents. While different modes of administration, using either gene therapy or peptide therapy (including intravenous or intraperitoneal injections of viral vectors; more targeted routes such as intracerebral inoculations; and nasal sprays) have all been suggested, at present it is not clear which route might be most effective. In addition to directly increasing chaperone levels, pharmacological agents that indirectly impact either chaperone levels or activity could also be effective, albeit with possible off-target effects.

In summary, while the last decade has resulted in an explosion of information on the mechanism of action of many cellular chaperones, we are only now beginning to understand the biochemical mechanisms involved in extracellular proteostasis. Future work in this area will provide us with a complete appreciation of the likely many redundant mechanisms brain cells employ to carry out extracellular proteostasis over the lifetime.

## Author Contributions

KC and TJ planned, edited, and wrote the majority of the manuscript. KC and TJ composed the figures and table. IL wrote, supervised, edited, and reviewed the manuscript. All authors contributed to the article and approved the submitted version.

## Conflict of Interest

The authors declare that the research was conducted in the absence of any commercial or financial relationships that could be construed as a potential conflict of interest.

## Footnotes

1 https://celltypes.brain-map.org/rnaseq/human_m1_10x

## Abbreviations

Abeta: amyloid beta
ACD: alpha crystallin domain
ACTH: adrenocorticotropic hormone
AD: Alzheimer’s disease
ALS: amyotrophic lateral sclerosis
APP: amyloid precursor protein
BiP: binding immunoglobulin protein
CAA: cerebral amyloid angiopathy
CRED: chaperone/receptor-mediated extracellular protein degradation
CSF: cerebrospinal fluid
ER: endoplasmic reticulum
ERAD: ER-associated degradation
FAD: familial Alzheimer’s disease
FBD: familial British dementia
FDD: familial Danish dementia
FTD: frontotemporal dementia
GRN: granulin
GWAS: genome-wide association studies
HSPGs: heparan-sulfated proteoglycans
HSPs: heat shock proteins
IDR: intrinsically disordered regions
NRBP1: nuclear receptor binding protein 1
PD: Parkinson’s disease
PP1: protein phosphatase 1
proPC2: pro-prohormone convertase 2
proSP-C: prosurfactant protein C
sHSP: small heat shock proteins
SNP: single nucleotide polymorphism
SPARC: secreted protein acidic and rich in cysteine
TDP-43: TAR DNA-binding protein 43
TFEB: transcription factor EB
UPR: unfolded protein response.
